# Host Adaptive Immune Status Regulates Expression of the Schistosome AMP-Activated Protein Kinase

**DOI:** 10.3389/fimmu.2018.02699

**Published:** 2018-11-21

**Authors:** Kasandra S. Hunter, Stephen J. Davies

**Affiliations:** Department of Microbiology and Immunology, F. Edward Hébert School of Medicine, Uniformed Services University of the Health Sciences, Bethesda, MD, United States

**Keywords:** *Schistosoma mansoni*, schistosome, adaptive immunity, AMP-activated protein kinase, energy metabolism, development

## Abstract

Schistosomes exhibit profound developmental adaptations in response to the immune status of their mammalian host, including significant attenuation of parasite growth, development and reproduction in response to deficits in host adaptive immunity. These observations led us to hypothesize that schistosomes regulate the utilization of energy resources in response to immunological conditions within the host. To test this hypothesis, we identified and characterized the *Schistosoma mansoni* AMP-activated protein kinase (AMPK), a heterotrimeric enzyme complex that is central to regulating energy metabolism at the cellular and organismal level in eukaryotes. We show that expression of the catalytic α subunit is developmentally regulated during the parasite life cycle, with peak expression occurring in adult worms. However, the protein is present and phosphorylated in all life cycle stages examined, suggesting a need for active regulation of energy resources throughout the life cycle. In contrast, transcription of the AMPK α gene is down-regulated in cercariae and schistosomula, suggesting that the protein in these life cycle stages is pre-synthesized in the sporocyst and that expression must be re-initiated once inside the mammalian host. We also show that schistosome AMPK α activity in adult worms is sensitive to changes in the parasite's environment, suggesting a mechanism by which schistosome metabolism may be responsive to host immune factors. Finally, we show that AMPK α expression is significantly down-regulated in parasites isolated from immunodeficient mice, suggesting that modulation of parasite energy metabolism may contribute to the attenuation of schistosome growth and reproduction in immunodeficient hosts. These findings provide insights into the molecular interactions between schistosomes and their vertebrate hosts and suggest that parasite energy metabolism may represent a novel target for anti-schistosome interventions.

## Introduction

Parasitic platyhelminths of the genus *Schistosoma* are the causative agents of schistosomiasis. It is estimated that at least 230 million individuals worldwide suffer from schistosomiasis (1–3), while ~600 million more are at risk of infection (4, 5). Most human schistosomiasis is attributed to just three parasite species—*S. haematobium, S. japonicum*, and *S. mansoni*. In the case of *S. japonicum* and *S. mansoni*, adult male and female worms reside in the mesenteric vasculature and produce hundreds or thousands of eggs per pair each day (6). Eggs either pass into host feces and exit into the environment to continue the parasite life cycle, or are trapped in host tissues where they cause inflammation and potentially life threatening complications. Virtually all the immunopathology associated with schistosomiasis, such as granulomas, fibrosis and subsequent portal hypertension, are solely due to the entrapment of parasite eggs in host tissue (7, 8). Furthermore, mouse models of schistosome infection have revealed that host immune factors can have profound effects on parasite development and reproduction. For example, in certain immunodeficient mice, schistosome growth and development is delayed and reproductive fitness is dramatically impaired (9, 10). We have shown that components of the host's adaptive immune system, particularly CD4^+^ T cells and T cell-derived cytokines, facilitate schistosome development and promote parasite egg production (10–12), indirectly through interactions with other cells such as monocytes and macrophages (13). Because of the immunopathological significance of schistosome eggs, targeting pathways that regulate egg production would be useful in decreasing global mortality and morbidity due to schistosomiasis.

While it is clear that schistosomes are auxotrophic for energy-producing substrates, such as glucose and fatty acids (14), and must acquire them from the host to sustain schistosome life and reproduction, it is unclear what intracellular processes control schistosome utilization of these resources, especially as it relates to egg production. Male and female schistosomes mainly engage in glycolysis to meet their bioenergetic needs (15–18), but catabolism of fatty acids via β-oxidation was also shown to be necessary for egg production by female schistosomes (6, 19). How environmental signals, such as those emanating from the host immune system, are integrated with metabolic regulation to control parasite bioprocesses such as egg production is unknown. Our previous work showed that pathways involved in cellular metabolism are altered in schistosomes isolated from immunodeficient mice (20). We found that expression and activity of schistosome protein kinase A (PKA), a key enzyme in eukaryotic cells with a central role in modulating cellular metabolic activity, is attenuated in schistosomes isolated from immunodeficient mice, correlating with reduced growth and reproductive fitness. Expression and activity of parasite PKA could be restored in immunodeficient mice by stimulating the host's immune system, suggesting a link between schistosome metabolic activity and the immune system of the mammalian host. Furthermore, restoration of PKA activity and expression correlated with restored parasite reproductive fitness, suggesting that control of cellular metabolism is not only important for schistosome egg production, but may also play a causal role in the attenuated developmental and reproductive phenotype of schistosomes in immunodeficient mice (20).

Evidence that PKA is involved in regulating schistosome reproduction prompted us to examine whether enzymes that are directly involved in specifically regulating energy metabolism are also implicated in regulating parasite reproductive activity. 5′-adenosine monophosphate-activated protein kinase (AMPK) is a heterotrimeric protein complex that plays a key role in cellular metabolic regulation and energy homeostasis. The trimeric AMPK complex is comprised of a catalytic α subunit, and β and γ subunits that perform regulatory functions (21). Within the protein kinase domain of the α subunit, a highly conserved threonine residue in the activation loop is characteristically phosphorylated when the protein is catalytically activated (22–24). The β subunit mainly acts as the scaffold holding the trimer together, and may also play a role in determining the subcellular localization of AMPK (25). The γ subunit contains four repeats of a cystathionine-β-synthase (CBS) domain that act to bind ATP, ADP, and AMP. When cellular ATP levels are low, AMP outcompetes ATP and ADP for binding the γ subunit, promoting the phosphorylation of the activation loop threonine in the α subunit kinase domain and leading to α subunit activation (22, 24, 26). Upon activation, AMPK activates pathways that produce ATP, and inhibits the activity of energy-consuming processes, in order to restore cellular equilibrium. Thus, these three subunits act together as a crucial nutrient sensor within the cell, integrating signals from the extracellular environment, transmitted via PKA and other upstream components, with cellular energy levels, to engage downstream effectors and processes that ensure energy homeostasis (23, 27).

In recent years, AMPK has received increasing attention, for its role in overall growth and reproduction in many organisms, and for its potential as a therapeutic target (28–33). In both yeast and *Drosophila*, AMPK is essential for regulating nutrient uptake and proper organismal growth, as the absence of AMPK abrogates nutrient uptake and storage and results in abnormal or delayed growth, and is sometimes lethal (34–36). In *Caenorhabditis elegans*, AMPK plays a fundamental role in formation of dauer larvae, a developmental stage triggered by limited environmental nutrient availability that exhibits decreased metabolic activity and rationing of energy stores—a state that is similar to the phenotype of schistosomes isolated from immunodeficient mice (37–39). Furthermore, AMPK is the target of new therapeutics for cell proliferative and metabolic diseases like cancer and diabetes, because exploiting the unique metabolic conditions in these diseases has proven a successful strategy for discriminating between diseased and normal tissue (29, 30, 33, 40). Because mounting evidence emphasizes the importance of AMPK to the survival of organisms of many species, elucidating the role of AMPK in schistosome survival, growth, and reproduction may reveal new opportunities to disrupt the parasite life cycle. Here, we present an initial characterization of the AMPK α subunit of *S. mansoni*. In addition to an analysis of *S. mansoni* AMPK α subunit expression and activity during the parasite life cycle, we provide evidence that AMPK α activity can be modulated in response to extrinsic factors, including those emanating from the host immune system.

## Materials and methods

### Ethics statement

All animal procedures were performed according to the current edition of the National Research Council's *Guide for the Care and Use of Laboratory Animals* (The National Academies Press, 2011) and pre-approved by the Institutional Animal Care and Use Committee at the Uniformed Services University of the Health Sciences, Assurance Number D16-00285 (A3448-01).

### Parasite materials

The *S. mansoni* (NMRI strain) life cycle was maintained using *Biomphalaria glabrata* (NMRI strain) snails and C57BL/6 mice as intermediate and definitive hosts, respectively. Mice were purchased from Jackson Laboratories. Snails infected with *S. mansoni* miracidia were provided by the Biomedical Research Institute. Infected snails were incubated under light for 1 h to promote the release of cercariae. To prepare schistosomula, cercariae were mechanically transformed by multiple passages through an emulsification needle. Cercarial heads were separated from the tails by swirling in deep Petri dishes. To obtain adult schistosomes, mice were infected with 150 cercariae each by tail skin exposure. At 8 weeks post-infection, adult worms were recovered from infected mice by portal vein perfusion. Eggs were isolated by homogenizing livers of infected mice and passing homogenates through stacked sieves of decreasing pore size (425, 180, 106, 45 μm). Eggs restricted by the smallest pore size were collected and cleaned for use. Miracidia were collected by hatching viable eggs in distilled water.

### Cloning and sequencing of *S. mansoni* AMPK α

Adult *S. mansoni* cDNA was used as the template for amplifying the full-length AMPK α cDNA sequence. To prepare cDNA, RNA was extracted from adult worm homogenates using RNAzolRT RNA Isolation Reagent. Crude RNA extracts were further purified using the RNeasy MinElute Cleanup Kit with DNase I digestion (Qiagen). One microgram RNA was used as the template for cDNA synthesis using the High-Capacity cDNA Reverse Transcription Kit (Applied Biosystems). A 533 bp fragment of the putative *S. mansoni* AMPK α, corresponding to nucleotides 668–1,201 of the predicted reference sequence Smp_142990 (mRNA XM_018799915.1) was amplified by conventional PCR using Accuprime Pfx Supermix (Thermo Fisher Scientific). The remaining full length cDNA sequence was then obtained using the RNA-ligase mediated rapid amplification of 5′ and 3′ cDNA ends (RACE) kit (Invitrogen). Products obtained by PCR and RACE were analyzed via 1.0% agarose gel, excised, and purified from the gel using the QIAquick Gel Extraction Kit (Qiagen). Purified fragments were then cloned into pCR-BluntII-TOPO vector using the Zero Blunt TOPO PCR Cloning Kit (Invitrogen) and used to transform One Shot Top10 Chemically Competent *E. coli* cells (Invitrogen). Transformants were plated on LB agar containing 50 μg/mL kanamycin and incubated at 37°C overnight. Single colonies were isolated and grown in LB overnight at 37°C with shaking. Cells were lysed and plasmid DNA was extracted using QIAprep Spin Miniprep Kit (Qiagen). Sequencing of plasmids was performed using the BigDye Terminator cycle sequencing kit (Thermo Fisher) and T7 primers. Sequence data was analyzed and contigs assembled using Geneious software, version 11.0.5 (Biomatters Ltd.).

### Preparation of parasite protein extracts

Parasite tissue was homogenized using an electric homogenizer in either RIPA lysis buffer or boiling 1X LDS sample buffer (NuPAGE LDS Sample Buffer, Invitrogen, NP0007) containing 10% 2-mercaptoethanol. Buffers were supplemented with phosphatase inhibitors (Halt Phosphatase Inhibitor Cocktail, Thermo Scientific) and protease inhibitors (Halt Protease Inhibitor Single-Use Cocktail, Thermo Scientific) to a final concentration of 1X. To obtain a homogenous solution, samples were further subjected to sonication. Samples homogenized in RIPA buffer were centrifuged at 16,100 g for 10 min to remove insoluble material. Samples were then boiled for 10 min and stored at −80°C for later use.

### Western blotting

Protein concentrations of parasite lysates were determined using Pierce 660 nm Protein Assay (Thermo Scientific) according to manufacturer's instructions. Ten percent Bis-Tris gels (NuPage) were loaded with 7 or 10 μg of protein per sample. Proteins were separated by SDS-PAGE in MES Buffer at 200 V for 1 h and transferred onto Invitrolon polyvinyl difluoride membranes (PVDF) (Novex) immediately after for 90 min at 25 V, 160 mA. Membranes were blocked for 30 min in TBS StartingBlock Blocking Buffer (Thermo Scientific) with 0.05% Tween-20. After blocking, membranes were incubated for 1 h with either anti-AMPK α-1, 2 rabbit polyclonal antibody [PA5-36045, Invitrogen; Research Resource Identifier (RRID) AB_2553341] diluted 1:500, anti-phospho-AMPK α (Thr172) (40H9) rabbit monoclonal antibody (2535S, Cell Signaling Technology) diluted 1:1,000, or anti-beta tubulin rabbit polyclonal antibody (Abcam) diluted 1:5,000. After primary antibody incubation, membranes were washed with 1X TBS Tween-20 buffer (TBST) (Thermo Scientific) and incubated for 1 h in goat anti-rabbit IgG secondary antibody, HRP (G21234, Invitrogen) diluted 1:2,000. All antibodies were diluted in blocking buffer with 0.05% Tween-20. To detect bound antibody, membranes were incubated in SuperSignal West Pico PLUS Chemiluminescent Substrate (Thermo Scientific). Membranes were imaged using an ImageQuant LAS 4000 system (GE Healthcare Life Sciences). Densitometry measurements were performed using ImageJ software (41). To conserve valuable samples for further analyses, in some experiments previously probed PVDF membranes were stripped by incubating in Restore Western Blot Stripping Buffer (Thermo Scientific) for 2 h at 37°C with agitation, to completely remove primary and secondary antibodies. After incubation in stripping buffer, the membranes were washed twice with TBST buffer at room temperature with agitation for 5 min and then blocked for 30 min in TBS StartingBlock Blocking Buffer with 0.05% Tween-20. After blocking, membranes were probed again with primary antibody as described above.

### Immunoprecipitation of AMPK α

AMPK α was immunoprecipitated from adult and schistosomula lysates using nProtein A Sepharose Fast Flow beads (GE Healthcare) according to the manufacturer's instructions. Before immunoprecipitation (IP), beads were washed in 1% NP-40 buffer (150 mM NaCl, 1% NP-40, 50 mM Tris-HCl pH 7.4, 1 mM EDTA). Upon final wash, a 50% slurry was prepared by mixing equal volumes of beads and NP-40 buffer. Adult worms were homogenized in NP-40 buffer. Schistosomula were lysed in boiling 1X LDS sample buffer. Lysates were precleared with nProtein A Sepharose Fast Flow 50% slurry by gently mixing for 1 h at 4°C. Precleared lysates were then centrifuged at 12,000 × g for 20 s and immunoprecipitation was performed using 50 μg parasite protein in a final volume of 250 μl NP-40 buffer. Either 1 μg anti-AMPK α or anti-phospho-AMPK α was added and the mixtures were incubated for 1 h at 4°C with gentle mixing. Lysate/antibody mixture was then incubated for 1 h at 4°C with 50 μl nProtein A Sepharose Fast Flow to precipitate immune complexes. Immunoprecipitated proteins were eluted by incubating immune complexed Sepharose beads in 30 μl 1X LDS sample buffer at 95°C for 3 min. Supernatants were analyzed by SDS-PAGE as described above.

### λ phosphatase sensitivity of anti-phospho AMPK α antibody

The specificity of the anti-phospho AMPK α for phosphorylated *S. mansoni* AMPK α was tested using Lambda Protein Phosphatase (Lambda PP) (New England BioLabs, P0753S). A 5 ml 1X solution of 10X NEBuffer was prepared by diluting in 1X TBST supplemented with 500 μl MnCl_2_. Twenty-five microliter of Lambda PP was added to each 5 ml preparation of 1X NEBuffer. PVDF membranes containing 10 μg of adult schistosome lysate per lane were incubated in 5 ml of the Lambda PP preparation either before or after incubation in anti-phospho-AMPK α diluted 1:1,000. Bound antibody was detected as described above. Phosphatase treated membranes were also incubated in anti-AMPK α 1, 2 and anti-beta tubulin as controls for protein loading.

### *In vitro* treatment of adult schistosomes with AMPK modulators

Immediately post -perfusion, fresh adult worms (5 pairs per well) were placed in the wells of 12 well tissue culture plates in 2 ml of modified Basch medium (Basal Medium Eagle, 2.8 mM glucose, 50 μg/ml hypoxanthine, 1X MEM vitamin solution, 1X MEM non-essential amino acids, 1X L-glutamine, 10 mM HEPES, 1X Pen/Strep, 10% FBS) containing either 100 or 200 μM forskolin or 0.1, 0.3, or 0.5 mM metformin. To determine the effects of no glucose, modified Basch medium was prepared without glucose. Schistosomes were incubated in their respective culture conditions for 2 h at 37°C. After incubation, worms were collected, lysates were prepared and analyzed by SDS-PAGE/Western blotting with anti-phospho-AMPK α, anti-AMPK α-1, 2, and anti-beta tubulin antibodies as described above.

### Detection of AMPK α gene expression in *S. mansoni* life cycle stages

To determine expression levels of AMPK α, quantitative real-time RT-PCR was performed on *S. mansoni* cDNA from every developmental stage. Total RNA from *S. mansoni* developmental stages was obtained from BEI Resources. Five hundred nanogram of RNA was used as template for cDNA synthesis reactions performed as previously described. A 64 bp fragment (nucleotide positions 751–815 of the *S. mansoni* AMPK α cDNA) was produced by PCR (TaqMan Fast Universal PCR Master Mix, ThermoFisher Scientific) from 1 μl cDNA reaction using the following primers: forward 5′-AGAATGATTACTGTGGACCCGATT-3′ and reverse 5′-AACCACGGATGTCGTCTGATTT-3′. A TaqMan MGB probe conjugated to a primer sequence situated at nucleotide position 776 (5′-AACGTGCAACCATAGAA-3′) was used to detect amplification of this fragment. A 557 bp fragment of Sm AMPK α (nt 388–944, of the *S. mansoni* AMPK α cDNA) was ligated into pCR-BluntII-TOPO vector (Invitrogen) as a control plasmid. Dilutions of this construct were used to generate a standard curve, from which AMPK α transcript copy number could be determined. All amplification reactions were performed and quantified using a 7500 Fast Real-Time PCR System (Applied Biosystems).

### Statistical analyses

For densitometry data derived from Western blots, the data in each experiment were normalized to that of one of the conditions tested, so that data from repeat experiments could be compared. Data from repeat experiments (at least two independent biological replicates were performed in every case) were considered to be paired repeated measures and were collectively analyzed using paired t tests. For quantitative PCR data, standard curves were used to calculate absolute transcript number in each RNA sample. For each source of RNA, at least three independent biological replicates were tested. The data for each RNA source were first tested for evidence of significant differences in variance, using *F*-tests. Because no significant differences in variance were found, the differences in transcript number between RNA sources were tested using parametric unpaired *t*-tests. All statistical analyses were performed using GraphPad Prism software, version 7.01 (GraphPad Software, Inc).

## Results

### Identification of the *Schistosoma mansoni* 5′-AMP-activated protein kinase α subunit

**Figure 1:**
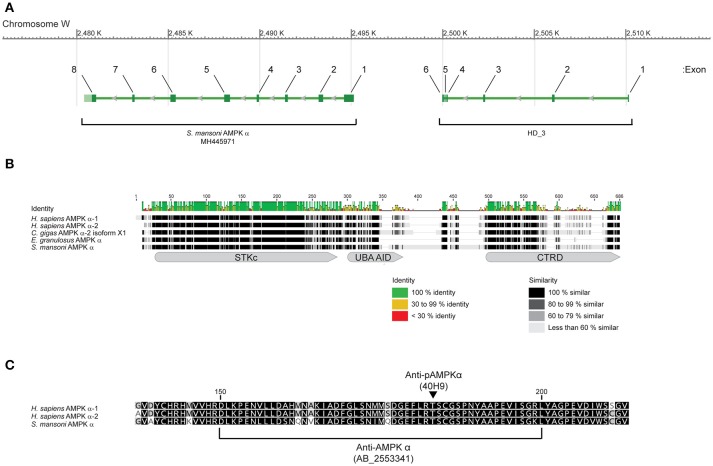
Sequence and structure of *S. mansoni* AMPK α. **(A)** Arrangement of the eight exons that contribute to the *S. mansoni* AMPK α mRNA MH445971, on the complementary strand of the W chromosome. Six exons that encode for a metal-dependent phosphohydrolase (HD_3) domain are located upstream, separated by 5 kb of intergenic sequence. **(B)** The amino acid sequence of *S. mansoni* AMPK α (MH445971) aligned with sequences of *Homo sapiens* AMPK α-1 (NP_006242) and AMPK α-2 (NP_006243), *Crassostrea gigas* AMPK α-2 (XP_011446066), and *Echinococcus granulosus* AMPK α (AER10553), showing sequence identity and similarity, and the approximate locations of the AMPK α serine/threonine kinase catalytic domain (STKc, CCD accession number cd14079), AMPK α UBA-like autoinhibitory domain (UBA AID, CCD accession number cd14336), and AMPK α C-terminal regulatory domain (CTRD, CCD accession number cd12122). Amino acid numbering refers to the consensus sequence for the alignment. **(C)** Alignment of *H. sapiens* AMPK α-1 (NP_006242) and AMPK α-2 (NP_006243), and *S. mansoni* AMPK α (MH445971), in the region recognized by a rabbit polyclonal antibody (RRID AB_2553341) raised against aa 150–200 of human AMPK α-1 (NP_006242). The square brackets denote the 50 aa sequence of NP_006242 used as the immunogen. The arrowhead indicates the position of the activation loop threonine that is phosphorylated upon activation, corresponding to Thr183 and Thr172 of NP_006242 and NP_006243, respectively, and Thr176 of the schistosome sequence (MH445971). When phosphorylated, this residue is recognized by the phospho-specific monoclonal antibody 40H9. Amino acid numbering corresponds to the sequence of NP_006242.

Annotation of the *S. mansoni* genome identified a putative single gene encoding for an AMPK α subunit, consisting of 14 exons, at locus Smp_142990 (Gene ID 8351843) on the complementary strand of the W chromosome, with predicted mRNA and protein products identified by accession numbers XM_018799915.1 and XP_018653781.1, respectively. While exhibiting high overall identity and similarity to AMPK α subunits of other species, this putative *S. mansoni* α subunit contained 830 amino acids, almost 300 residues more than AMPK α subunits of other organisms (Supplementary Figure 1A). Approximately 175 of the additional residues, encoded by the first six exons, are present in an N-terminal extension found in no other species (Supplementary Figure 1A). Comparison with the Conserved Domains Database (CCD) at the National Center for Biotechnology Information (NCBI) (42) revealed that the N-terminal extension contains a metal-dependent phosphohydrolase (HD_3) domain (CCD accession number pfam13023) (43), a feature not found in the AMPK α subunit of any other species we have examined. Using the putative mRNA sequence (XM_018799915.1) in a BLAST query of the expressed sequence tag (EST) database at NCBI (44), we identified many matching *S. mansoni* ESTs, with coverage across the entire length of the putative mRNA (Supplementary Figure 1B), suggesting the sequence is present in the *S. mansoni* transcriptome. However, no ESTs spanned the junction between the regions encoding the phosphohydrolase domain and the AMPK α subunit proper (Supplementary Figure 1B), suggesting the sequences may be derived from separate transcripts. To address this discrepancy, we used PCR and RACE to clone the *S. mansoni* AMPK α subunit cDNA from adult parasite cDNA. We succeeded in isolating a cDNA that corresponded to the eight 3' exons of the putative XM_018799915.1 mRNA (beginning at nucleotide position 538 of XM_018799915.1), but could find no evidence of the six 5′ exons that encode the HD_3 domain, despite exhaustive 5′ RACE and targeted PCR with specific primers. Together, these findings indicate that the HD_3-encoding exons do not contribute to the *S. mansoni* AMPK α subunit transcript, but rather represent a separate gene, located upstream on the complementary strand of the W chromosome and separated from the AMPK α gene by 5 kb of intergenic sequence (Figure 1A). We conclude that the cDNA we isolated (GenBank accession number MH445971) represents the complete *S. mansoni* AMPK α transcript.

The MH445971 cDNA contained a short 29-nucleotide 5′ untranslated region (UTR) followed by an open reading frame (ORF), beginning with a start methionine at nucleotide position 30, that encodes for a protein of 651 amino acids and predicted mass of 72 kDa. The predicted protein exhibited high identity and similarity to the AMPK α subunits of other organisms (Figure 1B). Analysis of the amino acid sequence using the CCD (42) revealed that the predicted protein contained all the canonical features of a typical AMPK α subunit (45): an AMPK α subunit serine/threonine kinase catalytic domain (CCD accession number cd14079) at amino acids 17-272 (*E* = 0e+00), an AMPK α UBA-like autoinhibitory domain (CCD accession number cd14336) at amino acids 289-353 (*E*-value = 1.08e-28), and an AMPK α C-terminal regulatory domain (CCD accession number cd12122) at amino acids 474-649 (*E*-value = 7.53e-36) (Figure 1B). Alignment of the *S. mansoni* AMPK α sequence with AMPK α subunits of other species revealed that the amino acid sequence was highly conserved throughout the kinase domain (Figure 1B), and particularly in the region of the activation loop (Figure 1C), where the conserved phosphorylation site of the human AMPK α subunits (Thr183 and Thr172 of human AMPK α-1 and AMPK α-2, respectively) was conserved at Thr176 of the schistosome protein (Figure 1C).

In addition to considerable lengths of conserved sequence, the *S. mansoni* AMPK α subunit also contained roughly 100 additional non-conserved amino acids, mostly clustered in the C-terminal half of the protein in two large insertions between the autoinhibitory and C-terminal regulatory domains (Figure 1B). These additional amino acids account for an appreciable difference in the predicted mass of the *S. mansoni* protein (72 kDa), as compared to human AMPK α (62 kDa).

### Detection of native *S. mansoni* AMPK α protein

**Figure 2:**
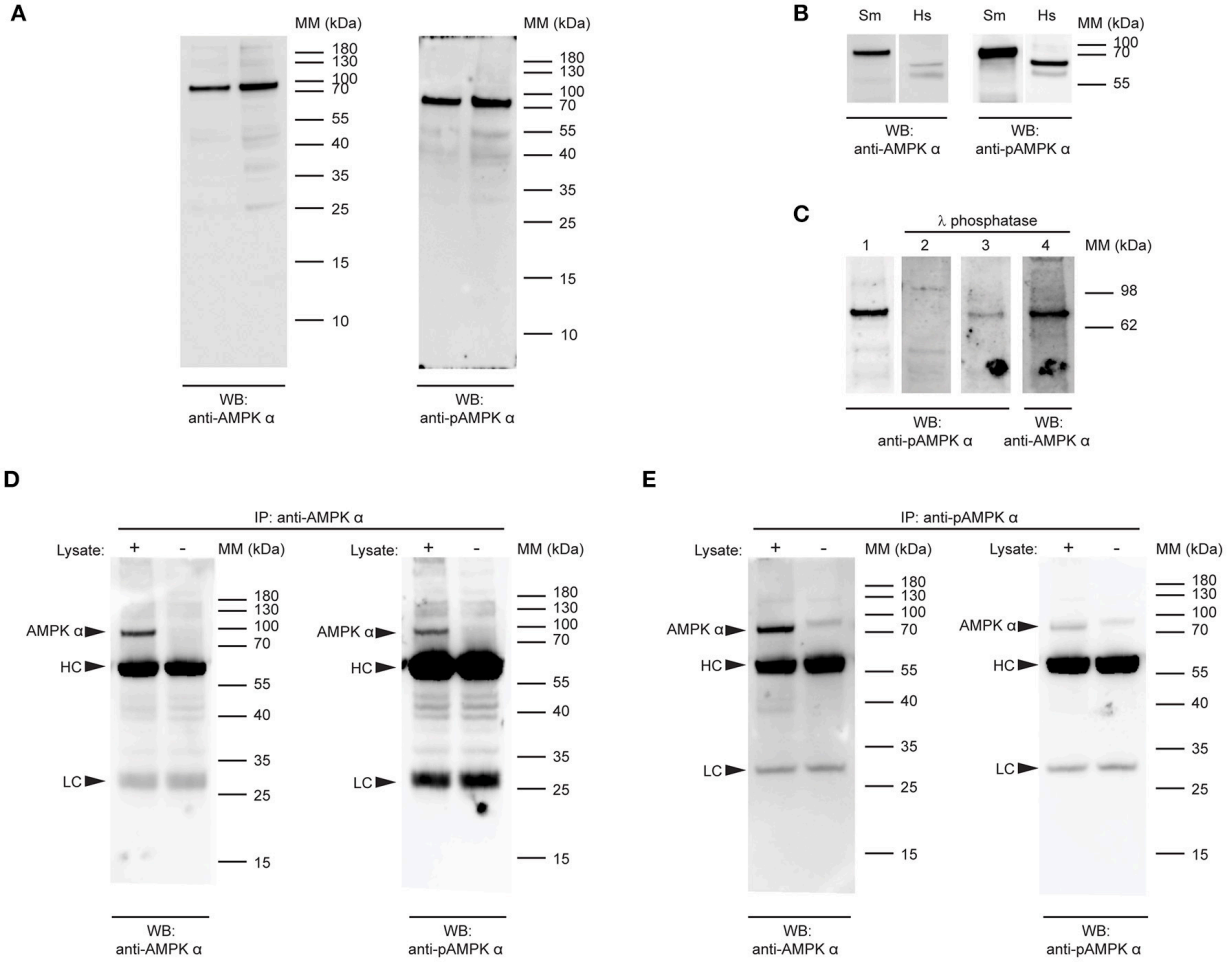
Detection of native *S.mansoni* AMPK α. **(A)** Duplicate samples of adult *S. mansoni* worm lysate were probed by Western blot with a polyclonal antibody raised against the catalytic domain of human AMPK α-1/2 (anti-AMPK α, RRID AB_2553341, left panel) and a phospho-specific monoclonal antibody that recognizes human AMPK α-1/2 when phosphorylated at Thr183/Thr172 (anti-pAMPK α, clone 40H9, right panel). **(B)** Lysates of adult *S.mansoni* worms (Sm) and human embryonic kidney cells (Hs), probed with anti-AMPK α (AB_2553341, left panel) and anti-pAMPK α (clone 40H9, right panel). **(C)** PVDF membranes bearing 10 μg adult *S. mansoni* worm lysate were probed with anti-pAMPK α (clone 40H9) either before (lane 1) or after treatment with λ protein phosphatase (lane 2). Lanes 3 and 4 are the same membrane as in lane 1, reprobed with anti-pAMPK α (lane 3) or anti-AMPK α (lane 4) after treatment with λ phosphatase. **(D,E)** AMPK α protein was immunoprecipitated from adult schistosome lysates using either anti-AMPK α **(D)** or anti-pAMPK α **(E)**. The immunoprecipitates were then probed by Western blot with anti-AMPK α (left panel) and anti-pAMPK α (right panel). Control immunoprecipitates where the lysate was omitted were also included. Arrowheads indicate the position of the schistosome AMPK α (AMPK α) and the immunoglobulin heavy chain (HC) and light chain (LC) of the immunoprecipitating antibody. WB, indicates the antibody used for Western blotting; IP, indicates the antibody used for immunoprecipitation; Positions of molecular mass (MM) markers, in kilodaltons (kDa), are indicated to the right of each panel.

The high level of identity between the schistosome and human AMPK α subunits in the region of the activation loop (Figure 1C) suggested that a rabbit polyclonal antibody raised against residues 150–200 of human AMPK α-1 (RRID AB_2553341) may cross-react with the schistosome AMPK α, as there are only five amino acid differences between the two proteins in this region (Figure 1C). Furthermore, the conservation of the phosphorylation site threonine (Thr176) and surrounding amino acids in the schistosome protein (Figure 1C) suggested that a monoclonal antibody that specifically binds human AMPK α-1/2 only when phosphorylated at Thr183/Thr172 (clone 40H9) may also bind specifically to phosphorylated schistosome AMPK α. When used to probe adult *S. mansoni* protein extracts by Western blot, both the anti-AMPK α polyclonal antibody and anti-phospho-AMPK α monoclonal antibody bound to a single band migrating slightly above the 70 kDa molecular mass marker (Figure 2A), consistent with the predicted molecular mass of the protein encoded by the cDNA we isolated (72 kDa). Western blots of adult schistosome and human embryonic kidney cell extracts probed with the two antibodies confirmed the expected difference in molecular mass between the AMPK α subunits of the two species (Figure 2B), the schistosome protein being ~10 kDa heavier due to the C-terminal insertions between the autoinhibitory and C-terminal regulatory domains (Figure 1B).

To confirm the specificity of the anti-phospho-AMPK α antibody for phosphorylated *S. mansoni* AMPK α, we tested whether λ phosphatase treatment of the protein was able to diminish antibody binding. When membranes containing *S. mansoni* adult worm lysate were treated with a λ protein phosphatase solution, anti-phospho-AMPK α antibody binding to the expected 72 kDa band was ablated or diminished (Figure 2C), while binding of the anti-AMPK α polyclonal antibody was not, providing evidence that the antibody binds specifically to a phosphorylated form of schistosome AMPK α.

To test whether the two antibodies were indeed binding to the same protein species, we performed reciprocal immunoprecipitation experiments, where we tested whether each antibody was able to bind to the protein immunoprecipitated by the other antibody. When immunoprecipitations of adult worm lysate performed with the anti-AMPK α polyclonal antibody were probed with the anti-AMPK α and anti-phospho-AMPK α antibodies, both antibodies bound to a 72 kDa band in the immunoprecipitates (Figure 2D). This band was not present when parasite lysate was omitted from the immunoprecipitate, confirming that the protein originated in the parasite lysate and was not derived from the preparation of immunoprecipitating antibody (Figure 2D). Likewise, when the anti-phospho-AMPK α antibody was used to immmunoprecipitate, both the anti-AMPK α and anti-phospho-AMPK α antibodies identified a 72 kDa band in the immunoprecipitations that was absent when the parasite lysate was omitted (Figure 2E). These data support the conclusion that both antibodies bind the same protein species in schistosome protein extracts.

### AMPK α expression and activation in *S. mansoni* larval stages

To determine if AMPK α protein is expressed and phosphorylated in larval stages of the *S. mansoni* life cycle, we used Western blotting to probe extracts of larval stages with the anti-AMPK α and anti-phospho-AMPK α antibodies. In preliminary experiments, we compared the suitability of different extraction methods to prepare lysates for Western blot analysis. Using cercariae as test material, we found that reactivity with both antibodies was lost altogether when parasites were lysed in RIPA buffer, and that a ladder of lower molecular mass bands was observed when the cercariae were lysed directly in 1 × sample buffer (data not shown), suggesting that the AMPK α was subject to rapid proteolytic degradation when extracted under these conditions, even though protease inhibitors were included in the extraction buffer. We found that the 72 kDa species was only extracted without significant degradation when the cercariae were lysed in boiling 1 × sample buffer, underscoring both the apparently labile nature of the schistosome AMPK α and the high protease content of cercariae. All larval stages were subsequently extracted using the boiling sample buffer method.

**Figure 3:**
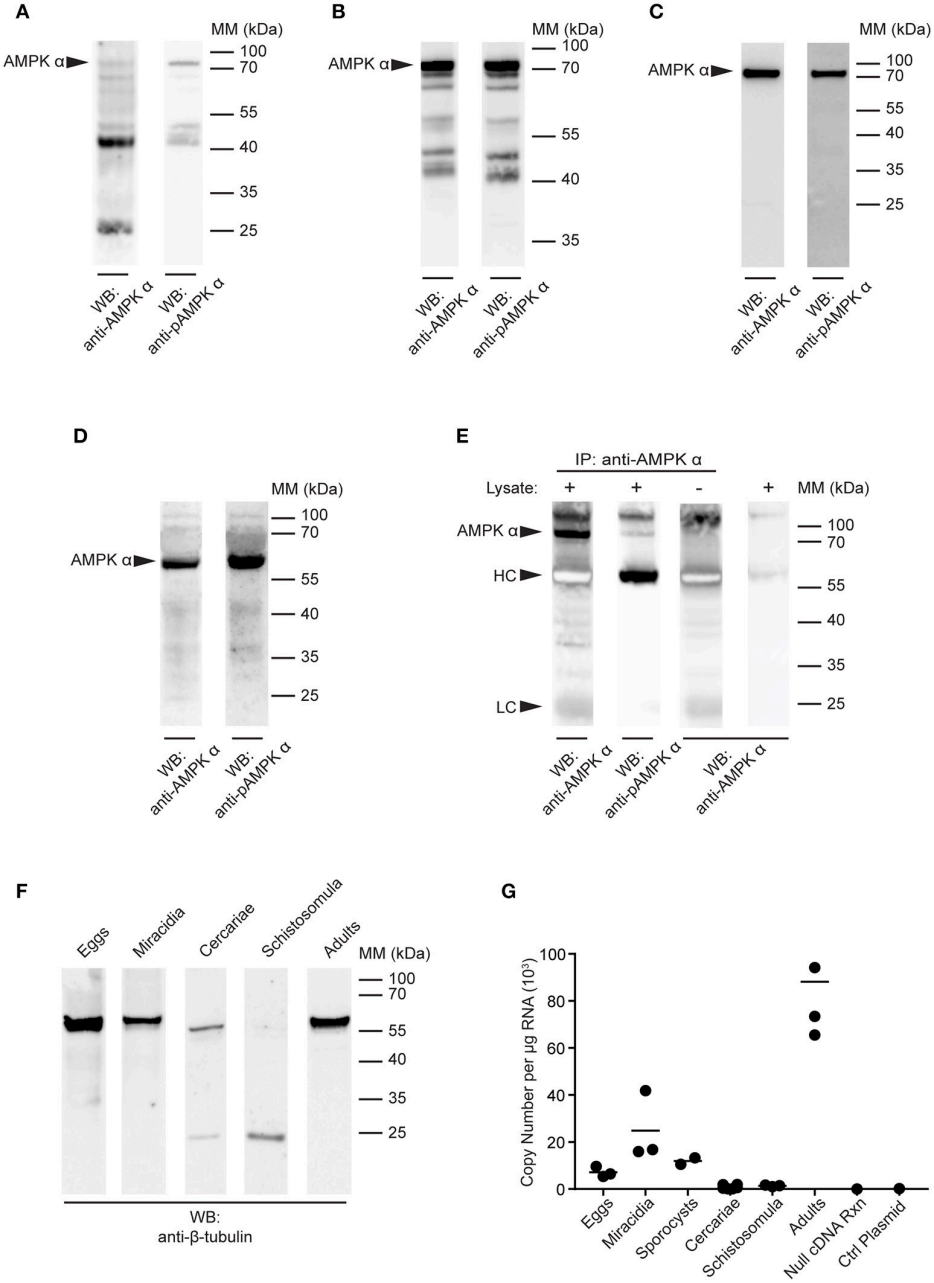
Detection of AMPK α in *S. mansoni* larval stages. **(A–D)** Lysates of *S. mansoni* eggs **(A)**, miracidia **(B)**, cercariae **(C)**, and schistosomula **(D)**, 30 min post-transformation were probed by Western blot using anti-AMPK α (left lane) and anti-pAMPK α (right lane) antibodies. **(E)** AMPK α was immunoprecipitated from schistosomula lysate using anti-AMPK α, then probed by Western blot using the anti-AMPK α and anti-pAMPK α antibodies. Control immnoprecipitations, where the either the lysate or the immunoprecipitating anti-AMPK α were omitted, were also probed with anti-AMPK α. Arrowheads indicate the position of the schistosome AMPK α (AMPK α) and the immunoglobulin heavy chain (HC) and light chain (LC) of the immunoprecipitating antibody. **(F)** Lysates of each larval stage and adult worms were probed by Western blot with an anti-β-tubulin antibody. **(G)** AMPK α transcripts in each *S. mansoni* developmental stage were quantified using quantitative real-time RT-PCR. Control reactions containing no cDNA template and a control plasmid lacking the AMPK α target sequence were also included. WB, indicates the antibody used for Western blotting; IP, indicates the antibody used for immunoprecipitation; Positions of molecular mass (MM) markers, in kilodaltons (kDa), are indicated to the right of each blot.

Lysates of *S. mansoni* eggs, miracidia, cercariae, and schistosomula (30 min post-transformation) were prepared using boiling sample buffer, equal amounts of protein were subjected to SDS-PAGE, and analyzed by Western blot. In blots of *S. mansoni* eggs, both antibodies produced only a weak signal at 72 kDa, with multiple lower molecular mass species now also detected, especially with the anti-AMPK α antibody, suggesting the protein may be subject to proteolytic degradation in this life cycle stage (Figure 3A). Blots of miracidia produced a clearer and more intense signal at 72 kDa, but also a pattern of lower molecular mass bands similar to that observed in eggs, most notably a doublet between the 55 and 40 kDa markers (Figure 3B). As miracidia only hatch from viable eggs, the miracidium extract used in Figure 3B is essentially enriched for the contents of viable eggs. The reactivity of the antibodies to egg extracts observed in Figure 3A may therefore be explained, at least in part, by the heterogeneous nature of egg preparations obtained from infected mouse livers, which contain dying, dead and non-viable eggs, in addition to viable eggs, and could therefore contain AMPK α in various states of degradation.

Western blotting of cercarial extracts produced a single 72 kDa band with both the anti-AMPK α and anti-phospho-AMPK α antibodies (Figure 3C), similar to that observed in adult worms (Figure 2). Immunoblotting of 30 min schistosomula extracts also revealed a single band (Figure 3D). However, in schistosomula the band appeared at a lower molecular mass (~60 kDa), apparently displaced by a large quantity of another unknown species running at approximately the same position as the 70 kDa mass marker (Figure 3D). To test whether this single reactive band in schistosomula was indeed the same species as that observed at 72 kDa in other life cycle stages (Figures 2, 3A–C), we immunoprecipitated the protein from schistosomula extracts using the anti-AMPK α antibody, to isolate the protein from the abundant species at 70 kDa. When the immunoprecipitates were probed with the anti-AMPK α and anti-phospho-AMPK α antibodies, we found that migration of the reactive band was restored to 72 kDa (Figure 3E), suggesting that its migration on direct blots was indeed distorted by the abundant unidentified species at 70 kDa.

As a loading control, equal amounts of the extracts of all life cycle stages were probed with an anti-β-tubulin antibody by Western blot. A band of approximately the expected mass (55 kDa) was detected in all life cycle stages, although in cercariae, and schistosomula, the signal was weaker, with evidence of lower molecular mass fragments, again emphasizing the difficulty of completely neutralizing the protease content of these life cycle stages when making protein extracts (Figure 3F).

To further investigate the expression of the AMPK α gene throughout the life cycle of *S. mansoni*, we used quantitative real-time RT-PCR to quantify AMPK α transcripts in each life cycle stage. Total RNA was extracted from every developmental stage, 500 ng of which was used as template for RT-PCR with primers and probe specific for the *S. mansoni* AMPK α cDNA. AMPK α message was detected in eggs, miracidia, sporocysts, and adult schistosomes, with the highest concentrations detected in adults (Figure 3G). In contrast, AMPK α transcript was nearly undetectable in cercariae and 30 min schistosomula.

### Schistosome AMPK α expression in adult female and male worms

**Figure 4:**
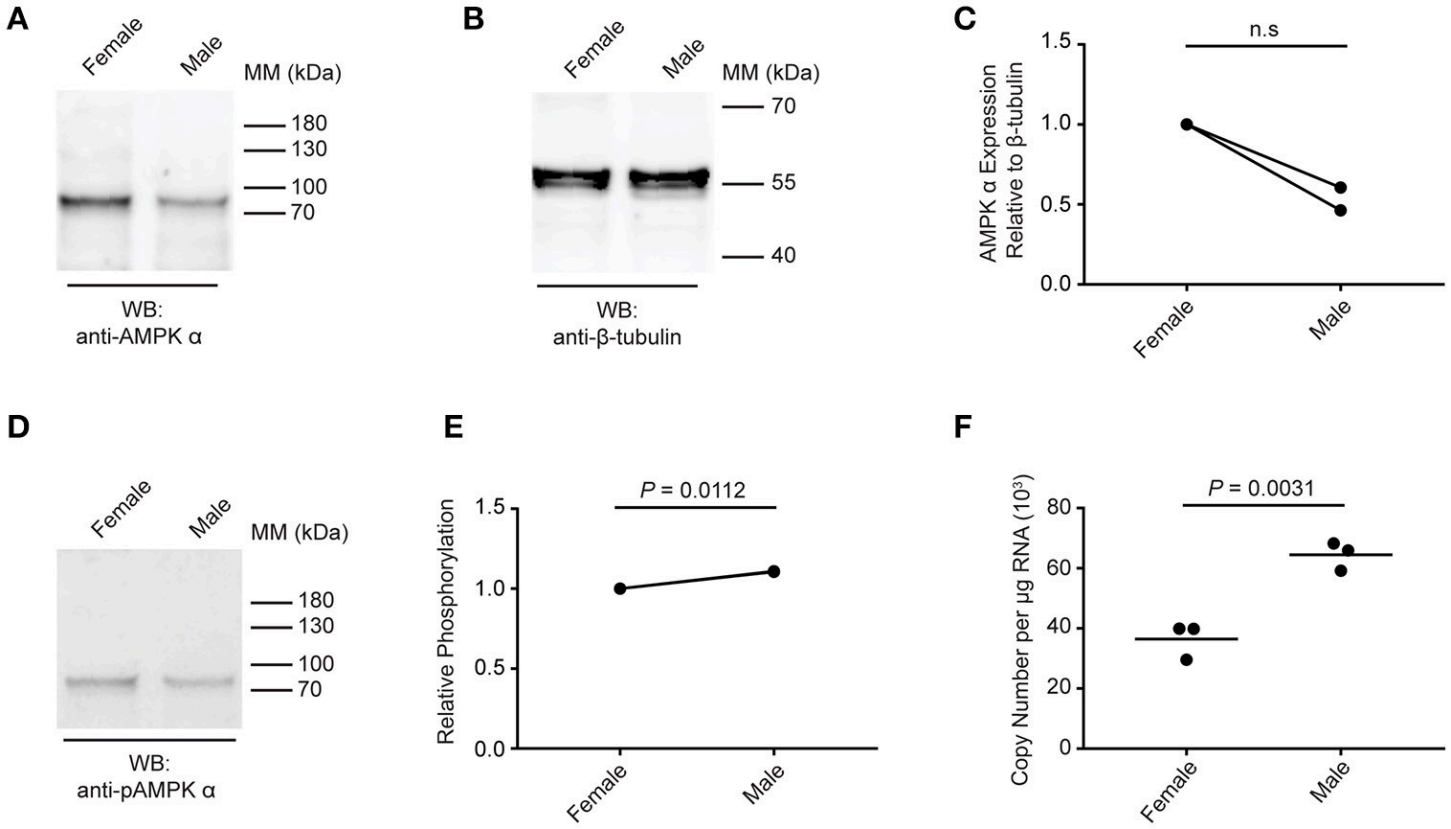
Expression and phosphorylation of AMPK α in female and male schistosomes. **(A)** Lysates of adult female and male schistosomes were probed by Western blot with anti-AMPK α antibodies. **(B)** Lysates of adult female and male schistosomes were probed by Western blot with anti-β-tubulin antibodies. **(C)** The intensity of the anti-AMPK α signal relative the anti-β-tubulin signal for each sample was determined, and the signals normalized to that obtained with female worms (results from two independent biological replicates are shown). **(D)** Lysates of adult female and male schistosomes were probed by Western blot with anti-pAMPK α antibody. **(E)** The intensity of the anti-pAMPK α signal relative the anti-AMPK α signal [from **(A)**] for each sample was determined, and the signals normalized to that obtained with female worms (results for two independent biological replicates are shown). **(F)** AMPK α transcripts in adult female and male *S. mansoni* were quantified using quantitative real-time RT-PCR. WB, indicates the antibody used for Western blotting; Positions of molecular mass (MM) markers, in kilodaltons (kDa), are indicated to the right of each blot.

Next, we compared the expression and phosphorylation of AMPK α in adult female and male schistosomes (Figure 4). Lysates were prepared from separated (i.e., previously paired) adult female and male *S. mansoni* and equal amounts of protein were analyzed by Western blot. Overall, female schistosomes appeared to contain more total AMPK α protein than male schistosomes, relative to the β-tubulin content of the extracts, although this difference was not significant when statistical analysis was performed on results from two biological replicates (Figures 4A–C). When AMPK α phosphorylation was assessed by Western blotting using the anti-phospho-AMPK α antibody, female and male schistosomes appeared to contain comparable amounts of phosphorylated AMPK α (Figure 4D). When phosphorylated AMPK α was normalized relative to total AMPK α protein, we found that AMPK α was phosphorylated to a marginally greater extent in males (Figure 4E). Data from two biological replicates revealed almost identical small increases in relative AMPK α phosphorylation in males, and statistical analysis of these data found the small difference was significant (Figure 4E). Finally, AMPK α gene expression in female and male worms was compared using PCR to measure transcript levels in RNA extracted from separated female and male worms. Adult male RNA contained significantly more AMPK α transcripts than adult female RNA (Figure 4F), but the difference was < 2-fold. From these limited data, we conclude that while some differences in AMPK α protein content and phosphorylation and AMPK α gene expression may exist between female and male schistosomes, the protein is likely expressed and phosphorylated in both sexes at broadly similar levels.

### Regulation of schistosome AMPK α phosphorylation in response to external stimuli

**Figure 5:**
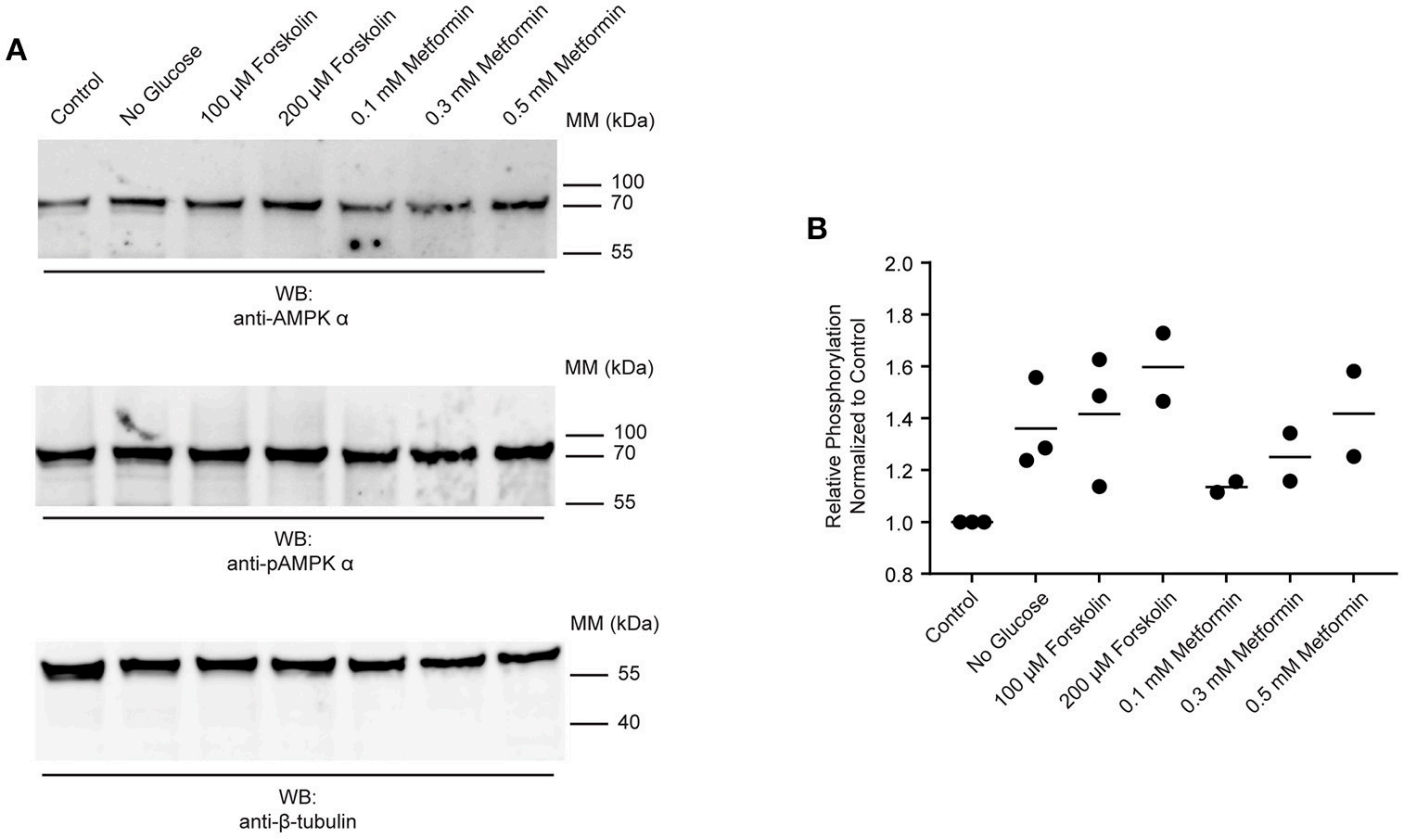
Responsiveness of schistosome AMPK α to external factors. **(A)** Freshly isolated adult schistosomes were incubated in control medium, medium lacking glucose, or medium supplemented with various concentrations of forskolin or metformin. After 2 h, lysates were prepared and equal amounts of protein were probed by Western blot, using anti-AMPK α antibody (top panel), anti-pAMPK α antibody (center panel), and anti-β-tubulin antibody (bottom panel). WB, indicates the antibody used for Western blotting; Positions of molecular mass (MM) markers, in kilodaltons (kDa), are indicated to the right of each blot. **(B)** Relative AMPK α phosphorylation under each condition was calculated by determining the ratio of the anti-pAMPK α signal to that of the anti-AMPK α signal, and then correcting for the intensity of the anti-β-tubulin signal. Relative AMPK α phosphorylation values were normalized relative those obtained with worms incubated in control medium. Data points from 2 to 3 independent biological replicates are shown.

The data presented in Figures 2, 4 suggest that AMPK α is constitutively phosphorylated in freshly isolated adult worms. We therefore sought to test whether further phosphorylation of schistosome AMPK α could be induced in adult worms immediately *ex vivo*, as evidence that the AMPK pathway in adult schistosomes is responsive to changes in the worm's environment. Adult worms were incubated in control medium, in medium lacking glucose, or in control medium supplemented with increasing concentrations of forskolin [an adenylate cyclase agonist that indirectly activates AMPK α by raising cellular cAMP concentrations (46, 47)] or metformin [a direct AMPK α agonist (29, 48–50)]. After 2 h of incubation under these conditions, lysates were prepared and equal amounts of total protein were analyzed by Western blot. The signal obtained with the anti-phospho-AMPK α antibody was normalized to that obtained with the anti-AMPK α antibody, which in turn was normalized to the signal obtained with the anti-β-tubulin antibody. Glucose deprivation consistently resulted in increased relative phosphorylation of AMPK α (Figures 5A,B). Likewise, exposure to different concentrations of forskolin and metformin resulted in increased phosphorylation of AMPK α (Figures 5A,B).

### *S. mansoni* AMPK α expression is attenuated in RAG^−/−^ mice

**Figure 6:**
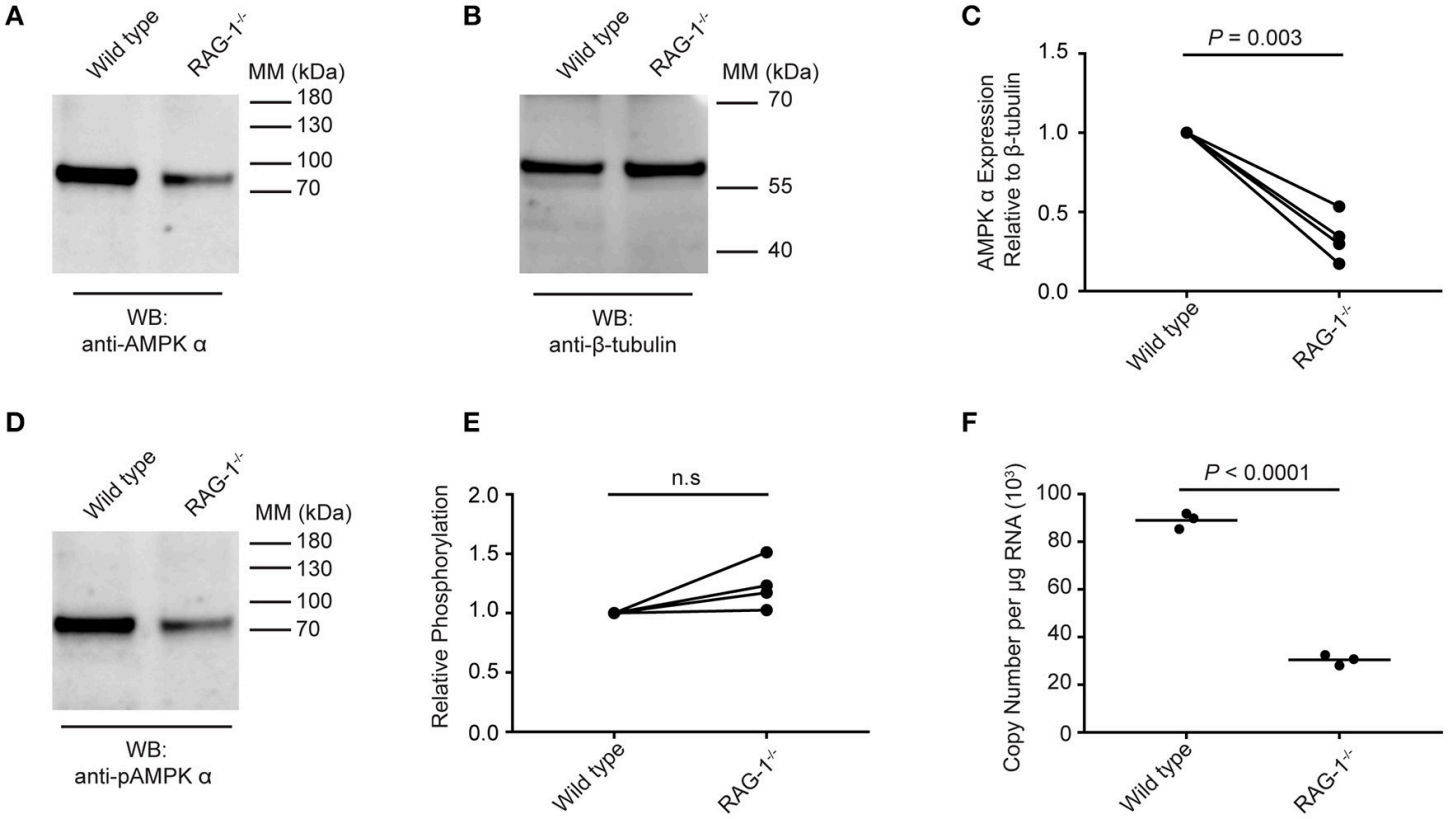
Expression and phosphorylation of schistosome AMPK α in RAG-1^−/−^ mice. Lysates were prepared from schistosomes recovered from wild type (C57BL/6) and RAG-1^−/−^ mice and probed by Western blot with anti-AMPK α **(A)** and anti-β-tubulin **(B)** antibodies. **(C)** The intensity of the anti-AMPK α signal relative the anti-β-tubulin signal for each sample was determined, and the signals normalized to that obtained with worms from wild type mice (displaying results from four independent biological replicates). **(D)** Lysates of schistosomes from wild type and RAG-1^−/−^ mice were probed by Western blot with anti-pAMPK α antibody. **(E)** The intensity of the anti-pAMPK α signal relative the anti-AMPK α signal [from **(A)**] for each sample was determined, and the signals normalized to that obtained with worms from wild type mice (displaying results from four independent biological replicates). **(F)** AMPK α transcripts in *S. mansoni* worms isolated from wild type and RAG-1^−/−^ mice were quantified using quantitative real-time RT-PCR. WB, indicates the antibody used for Western blotting; Positions of molecular mass (MM) markers, in kilodaltons (kDa), are indicated to the right of each blot.

Data presented in Figure 5 suggest that schistosome AMPK α activity can be modulated by extrinsic factors (e.g., environmental glucose availability, AMPK α modulators) in the parasites' environment. We therefore tested whether *S. mansoni* AMPK α phosphorylation was modulated in immunodeficient RAG-1^−/−^ mice, where schistosomes exhibit attenuated growth and reproductive fitness (10). Worms recovered from wild type and RAG-1^−/−^ mice were assessed for AMPK α expression and phosphorylation by Western blot. Probing with the anti-AMPK α antibody, we found that worms isolated from wild type mice contained approximately twice as much total AMPK α protein as worms from RAG-1^−/−^ mice, relative to the β-tubulin content of the lysates (Figures 6A,B). This decrease in total AMPK α protein content in worms from RAG-1^−/−^ mice was found to be reproducible and statistically significant when data from four biological replicates were analyzed (Figure 6C). When the lysates were probed with the anti-phospho-AMPK α antibody, worms from wild type mice again contained more phosphorylated AMPK α than worms from RAG-1^−/−^ mice (Figure 6D), mirroring the difference in total AMPK α content of the worms. However, when the levels of phosphorylated AMPK α were normalized relative to the total AMPK α protein content of the lysates, we found that relative phosphorylation of schistosome AMPK α was similar in both host genotypes, with no significant difference evident when data from four biological replicates were analyzed (Figure 6E). When the AMPK α mRNA content of the worms was compared by PCR, we found that worms from RAG-1^−/−^ mice contained less than half the concentration of AMPK α transcripts as worms from wild type mice (Figure 6F). Thus, expression of AMPK α in worms from RAG-1^−/−^ mice is down-regulated at the level of both mRNA and protein (Figures 6A–C,F), but levels of relative AMPK α activity are similar to those in wild type mice (Figure 6E).

## Discussion

Overall, schistosome AMPK α exhibits high homology to AMPK catalytic subunits of other organisms, with the exception of a 100 amino acid insertion in the C-terminal half of the schistosome sequence that is not found in the AMPK α of any other organism. The impact of this insertion on the tertiary structure of *S. mansoni* AMPK α will require specific structural and functional analyses, but we hypothesize that the greater distance between the protein's C-terminal regulatory and N-terminal kinase/autoinhibitory domains may introduce greater flexibility into the molecule, and increase the potential for these domains to engage in intermolecular interactions with other proteins. If this unique sequence does have functional significance, it may have the potential to serve as a unique target to interrupt AMPK activity in schistosomes, without detrimentally affecting the human ortholog.

Our Western blotting experiments show that AMPK α protein is present and active in all life cycle stages we examined, likely reflecting a need for active energy homeostasis throughout the schistosome life cycle. In contrast, AMPK α transcripts were detectable in all life cycle stages examined, with the notable exception of cercariae and 30 min schistosomula. Previous studies have shown that cercariae generally do not actively engage in transcription, with the exception of a subset of genes that are mostly expressed in cercarial tails (51, 52), and that transcription of most genes is reinitiated once the parasite enters the mammalian host (53). Our data suggest that the AMPK α gene is also regulated in this manner, such that AMPK proteins are prepackaged into cercariae before leaving the molluscan host, and the AMPK α gene is not actively expressed again until the parasite enters the mammalian host. We found that AMPK α transcripts were most abundant in adult worms, so at some point between cercarial skin penetration and adulthood, dramatic up-regulation of AMPK α transcription must occur. Studies are underway to identify the exact time point at which this occurs. The presence of AMPK α protein, but not transcripts, during the cercariae/schistosomulum stage of the life cycle suggests this may be an opportune point to target schistosome AMPK α, when the parasite is unable to synthesize new protein.

The results of our *in vitro* experiments showed that AMPK α activity is responsive to changes in the parasites' environment, suggesting AMPK α could potentially play a role in sensing host factors. The impaired growth and reduced reproductive fitness of schistosomes infecting immunodeficient mice is well documented and is an obvious developmental response to a change in host factors (9, 10, 13, 54). We were therefore curious to explore whether any changes in AMPK α expression or activity correlated with this developmental adaptation. Here we found that this attenuated developmental phenotype coincided with a reduction in overall AMPK α protein levels, while the AMPK α that is expressed is phosphorylated to a similar extent to AMPK α in parasites from immunocompetent hosts. One possible explanation for why schistosome growth and reproduction are attenuated in immunodeficient hosts is that the parasites are unable to secure sufficient essential host resources to sustain normal growth and development in an immunocompromised environment. However, we have been unable to show that the availability of fatty acids or glucose are reduced in immunodeficient hosts (Hunter and Davies, unpublished), and the consistent level of AMPK α phosphorylation in worms from immunocompetent and immunodeficient mice argues that the parasites are not struggling to meet their bioenergetic needs in an immunodeficient environment. If schistosomes growing in immunodeficient mice were experiencing metabolic stress, we would expect AMPK α phosphorylation to be increased in these worms as they engage mechanisms that serve to restore cellular ATP levels, but this is not the case. While we cannot rule out the possibility that a reduction in the availability of some other essential host resource accounts for the impaired development of schistosomes in immunodeficient mice, our data argue that impeded acquisition of energy substrates is not the cause of the attenuated parasite development in this context.

An alternative hypothesis for the attenuated developmental phenotype of schistosomes in immunodeficient mice postulates that (i) the parasites require a key but as yet unidentified developmental signal from the host to develop and reproduce at normally rates, and (ii) that this developmental signal either originates from the host's immune system or is otherwise produced as a result of the host immune response. Hence, in immunodeficient mice the putative developmental signal is not expressed and the parasites revert to an alternative developmental program, where development and reproduction proceeds but at a slower pace than normal (10, 54). Our data showing that schistosome AMPK α transcripts and protein are expressed at a lower level in immunodeficient hosts could be consistent with an alternative developmental state, where bioenergetic demands are different and AMPK α expression is regulated accordingly. Indeed, AMPK α transcript levels in worms from RAG-1^−/−^ mice are more similar to those found in miracidia than in adult worms from wild type mice. These results closely resemble the findings we previously published concerning schistosome PKA, which is also expressed at significantly reduced levels in immunodeficient hosts (20). Together these results suggest that schistosome metabolism is subject to significant modification in response to host immune status. While it is unlikely that schistosomes, under natural conditions, encounter hosts as profoundly immunodeficient as the RAG-1^−/−^ mice used in our studies, causes of secondary or acquired immunodeficiency, such as malnutrition and coinfection with human immunodeficiency virus and other pathogens, are prevalent within the geographic range of schistosomes (55–59). Hence, the ability to modify parasite metabolic activity in response to alterations in host immune function may confer a selective advantage.

The downstream effects of AMPK activation are numerous. In mammals and other eukaryotes, phosphorylation of AMPK α ultimately leads to the upregulation of catabolic processes, including fatty acid oxidation and glycolysis, and mitochondrial biogenesis and autophagy (22, 24, 26). Further metabolic analyses will be required to determine whether the activation of schistosome AMPK α results in the same consequences. Given that glycolysis and fatty acid oxidation have both been shown to be essential for parasite survival and reproduction (6, 16–19), elucidating the connection between host immune signals, AMPK, and energy generating processes in schistosomes is of considerable interest. The influence of schistosome AMPK on parasite glucose transporter expression is also being explored. In mammals, the mechanism by which AMPK activation upregulates glycolysis is in part fueled by an increase in glucose uptake via enhanced expression of glucose transporters (22, 60, 61). Identifying schistosome metabolic processes regulated by AMPK and mechanisms by which this occurs may reveal new opportunities for disrupting parasite growth, development and reproduction.

## Data availability statement

The nucleotide sequence of the *S. mansoni* AMPK α subunit is available as GenBank accession number MH445971. All other relevant data for this study are included in the manuscript and the Supplementary Files.

## Author contributions

KH and SD conceived and designed, analyzed, and wrote the experiments described herein. KH collected the data.

### Conflict of interest statement

The authors declare that the research was conducted in the absence of any commercial or financial relationships that could be construed as a potential conflict of interest.

## References

[1] Fenwick A Savioli L Engels D Robert Bergquist N Todd MH. Drugs for the control of parasitic diseases: current status and development in schistosomiasis. Trends Parasitol. (2003) 19:509–15. 10.1016/j.pt.2003.09.005 14580962

[2] Fenwick A. The global burden of neglected tropical diseases. Public Health (2012) 126:233–6. 10.1016/j.puhe.2011.11.015 22325616

[3] Colley DG Bustinduy AL Secor WE King CH. Human schistosomiasis. Lancet (2014) 383:2253–64. 10.1016/S0140-6736(13)61949-2 24698483 PMC4672382

[4] Adenowo AF Oyinloye BE Ogunyinka BI Kappo AP. Impact of human schistosomiasis in sub-Saharan Africa. Braz J Infect Dis. (2015) 19:196–205. 10.1016/j.bjid.2014.11.004 25636189 PMC9425372

[5] Dawaki S Al-Mekhlafi HM Ithoi I Ibrahim J Abdulsalam AM Ahmed A. Prevalence and risk factors of schistosomiasis among hausa communities in Kano State, Nigeria. Rev Inst Med Trop Sao Paulo (2016) 58:54. 10.1590/S1678-9946201658054 27410914 PMC4964323

[6] Pearce EJ Huang SC. The metabolic control of schistosome egg production. Cell Microbiol. (2015) 17:796–801. 10.1111/cmi.12444 25850569 PMC4867551

[7] Doenhoff MJ Hassounah O Murare H Bain J Lucas S The schistosome egg granuloma: immunopathology in the cause of host protection or parasite survival? Trans R Soc Trop Med Hyg. (1986) 80:503–14. 10.1016/0035-9203(86)90126-4 3492792

[8] Gryseels B Polman K Clerinx J Kestens L. Human schistosomiasis. Lancet (2006) 368:1106–18. 10.1016/S0140-6736(06)69440-3 16997665

[9] Harrison R Doenhoff M. Retarded development of *Schistosoma mansoni* in immunosuppressed mice. Parasitology (1983) 86:429–38. 10.1017/S0031182000050629 6877869

[10] Davies SJ Grogan JL Blank RB Lim KC Locksley RM McKerrow JH Modulation of blood fluke development in the liver by hepatic CD4+ lymphocytes. Science (2001) 295:1358–61. 10.1126/science.1064462 11701932

[11] Davies SJ McKerrow JH. Developmental plasticity in schistosomes and other helminths. Int J Parasitol. (2003) 33:1277–84. 10.1016/S0020-7519(03)00161-9 13678642 PMC2891239

[12] Riner DK Ferragine CE Maynard SK Davies SJ. Regulation of innate responses during pre-patent schistosome infection provides an immune environment permissive for parasite development. PLoS Pathog. (2013) 9:e1003708. 10.1371/journal.ppat.1003708 24130499 PMC3795041

[13] Lamb EW Walls CD Pesce JT Riner DK Maynard SK Crow ET. Blood fluke exploitation of non-cognate CD4+ T cell help to facilitate parasite development. PLoS Pathog. (2010) 6:e1000892. 10.1371/journal.ppat.1000892 20442785 PMC2861709

[14] Berriman M Haas BJ LoVerde PT Wilson RA Dillon GP Cerqueira GC. The genome of the blood fluke *Schistosoma mansoni*. Nature (2009) 460:352–8. 10.1038/nature08160 19606141 PMC2756445

[15] Bueding E. Carbohydrate metabolism of *Schistosoma mansoni*. J Gen Physiol. (1950) 33:475–95. 10.1085/jgp.33.5.475 15422103 PMC2147213

[16] Shapiro TA Talalay P. S*chistosoma mansoni*:mechanisms in regulation of glycolysis. Exp Parasitol. (1982) 54:379–90. 10.1016/0014-4894(82)90047-9 7151946

[17] Skelly PJ Da'dara AA Li XH Castro-Borges W Wilson RA. Schistosome feeding and regurgitation. PLoS Pathog. (2014) 10:e1004246. 10.1371/journal.ppat.1004246 25121497 PMC4133383

[18] You H Stephenson RJ Gobert GN McManus DP. Revisiting glucose uptake and metabolism in schistosomes: new molecular insights for improved schistosomiasis therapies. Front Genet. (2014) 5:176. 10.3389/fgene.2014.00176 24966871 PMC4052099

[19] Huang SC Freitas TC Amiel E Everts B Pearce EL Lok JB. Fatty acid oxidation is essential for egg production by the parasitic flatworm *Schistosoma mansoni*. PLoS Pathog. (2012) 8:e1002996. 10.1371/journal.ppat.1002996 23133378 PMC3486914

[20] Swierczewski BE Davies SJ. Developmental regulation of protein kinase A expression and activity in *Schistosoma mansoni*. Int J Parasitol. (2010) 40:929–35. 10.1016/j.ijpara.2010.01.001 20097200 PMC2875359

[21] Lage R Dieguez C Vidal-Puig A Lopez M. AMPK: a metabolic gauge regulating whole-body energy homeostasis. Trends Mol Med. (2008) 14:539–49. 10.1016/j.molmed.2008.09.007 18977694

[22] Hardie DG Ross FA Hawley SA. AMPK: a nutrient and energy sensor that maintains energy homeostasis. Nat Rev Mol Cell Biol. (2012) 13:251–62. 10.1038/nrm3311 22436748 PMC5726489

[23] Hardie DG. AMPK–sensing energy while talking to other signaling pathways. Cell Metab. (2014) 20:939–52. 10.1016/j.cmet.2014.09.013 25448702 PMC5693325

[24] Hardie DG Schaffer BE Brunet A. AMPK: an energy-sensing pathway with multiple inputs and outputs. Trends Cell Biol. (2016) 26:190–201. 10.1016/j.tcb.2015.10.013 26616193 PMC5881568

[25] Inoki K Kim J Guan KL. AMPK and mTOR in cellular energy homeostasis and drug targets. Annu Rev Pharmacol Toxicol. (2012) 52:381–400. 10.1146/annurev-pharmtox-010611-134537 22017684

[26] Alexander A Walker CL. The role of LKB1 and AMPK in cellular responses to stress and damage. FEBS Lett. (2011) 585:952–7. 10.1016/j.febslet.2011.03.010 21396365

[27] Mihaylova MM Shaw RJ. The AMPK signalling pathway coordinates cell growth, autophagy and metabolism. Nat Cell Biol. (2011) 13:1016–23. 10.1038/ncb2329 21892142 PMC3249400

[28] Zhang BB Zhou G Li C. AMPK: an emerging drug target for diabetes and the metabolic syndrome. Cell Metab. (2009) 9:407–16. 10.1016/j.cmet.2009.03.012 19416711

[29] Hardie DG. AMPK: a target for drugs and natural products with effects on both diabetes and cancer. Diabetes (2013) 62:2164–72. 10.2337/db13-0368 23801715 PMC3712072

[30] Coughlan KA Valentine RJ Ruderman NB Saha AK. AMPK activation: a therapeutic target for type 2 diabetes? Diabetes Metab Syndr Obes. (2014) 7:241–53. 10.2147/DMSO.S43731 25018645 PMC4075959

[31] Liu X Chhipa RR Nakano I Dasgupta B. The AMPK inhibitor compound C is a potent AMPK-independent antiglioma agent. Mol Cancer Ther. (2014) 13:596–605. 10.1158/1535-7163.MCT-13-0579 24419061 PMC3954437

[32] Yao F Zhang M Chen L. 5'-Monophosphate-activated protein kinase (AMPK) improves autophagic activity in diabetes and diabetic complications. Acta Pharm Sin B (2016) 6:20–5. 10.1016/j.apsb.2015.07.009 26904395 PMC4724658

[33] Day EA Ford RJ Steinberg GR. AMPK as a therapeutic target for treating metabolic diseases. Trends Endocrinol Metab. (2017) 28:545–60. 10.1016/j.tem.2017.05.004 28647324

[34] Bland ML Lee RJ Magallanes JM Foskett JK Birnbaum MJ. AMPK supports growth in *Drosophila* by regulating muscle activity and nutrient uptake in the gut. Dev Biol. (2010) 344:293–303. 10.1016/j.ydbio.2010.05.010 20478298 PMC2909368

[35] Garcia-Salcedo R Lubitz T Beltran G Elbing K Tian Y Frey S. Glucose de-repression by yeast AMP-activated protein kinase SNF1 is controlled via at least two independent steps. FEBS J. (2014) 281:1901–17. 10.1111/febs.12753 24529170

[36] Hsu JW Chen KJ Lee FJ. Snf1/AMP-activated protein kinase activates Arf3p to promote invasive yeast growth via a non-canonical GEF domain. Nat Commun. (2015) 6:7840. 10.1038/ncomms8840 26198097 PMC4525183

[37] Murphy CT McCarroll SA Bargmann CI Fraser A Kamath RS Ahringer J. Genes that act downstream of DAF-16 to influence the lifespan of *Caenorhabditis elegans*. Nature (2003) 424:277–83. 10.1038/nature01789 12845331

[38] Apfeld J O'Connor G McDonagh T DiStefano PS Curtis R. The AMP-activated protein kinase AAK-2 links energy levels and insulin-like signals to lifespan in *C*. elegans. Genes Dev. (2004) 18:3004–9. 10.1101/gad.1255404 15574588 PMC535911

[39] Uno M Nishida E. Lifespan-regulating genes in *C*. elegans. NPJ Aging Mech Dis. (2016) 2:16010. 10.1038/npjamd.2016.10 28721266 PMC5514992

[40] Motoshima H Goldstein BJ Igata M Araki E. AMPK and cell proliferation–AMPK as a therapeutic target for atherosclerosis and cancer. J Physiol. (2006) 574(Pt 1):63–71. 10.1113/jphysiol.2006.108324 16613876 PMC1817805

[41] Schneider CA Rasband WS Eliceiri KW. NIH Image to ImageJ: 25 years of image analysis. Nat Methods (2012) 9:671–5. 10.1038/nmeth.2089 22930834 PMC5554542

[42] Marchler-Bauer A Bo Y Han L He J Lanczycki CJ Lu S. CDD/SPARCLE: functional classification of proteins via subfamily domain architectures. Nucleic Acids Res. (2017) 45:D200–D203. 10.1093/nar/gkw1129 27899674 PMC5210587

[43] Aravind L Koonin EV. The HD domain defines a new superfamily of metal-dependent phosphohydrolases. Trends Biochem Sci. (1998) 23:469–72. 10.1016/S0968-0004(98)01293-6 9868367

[44] Johnson M Zaretskaya I Raytselis Y Merezhuk Y McGinnis S Madden TL. NCBI BLAST: a better web interface. Nucleic Acids Res. (2008) 36:W5–9. 10.1093/nar/gkn201 18440982 PMC2447716

[45] Xiao B Sanders MJ Underwood E Heath R Mayer FV Carmena D. Structure of mammalian AMPK and its regulation by ADP. Nature (2011) 472:230–3. 10.1038/nature09932 21399626 PMC3078618

[46] Yin W Mu J Birnbaum MJ. Role of AMP-activated protein kinase in cyclic AMP-dependent lipolysis In 3T3-L1 adipocytes. J Biol Chem. (2003) 278:43074–80. 10.1074/jbc.M308484200 12941946

[47] Egawa M Kamata H Kushiyama A Sakoda H Fujishiro M Horike N. Long-term forskolin stimulation induces AMPK activation and thereby enhances tight junction formation in human placental trophoblast BeWo cells. Placenta (2008) 29:1003–8. 10.1016/j.placenta.2008.09.008 18950855

[48] Musi NH Hirshman MF Nygren J Svandfelt M Bavenholm P Rooyackers O Metformin increases AMP-activated protein kinase activity in skeletal muscles of subjects with type 2 *Diabetes*. Diabetes (2002) 51:2074–81. 10.2337/diabetes.51.7.2074 12086935

[49] Foretz M Guigas B Bertrand L Pollak M Viollet B. Metformin: from mechanisms of action to therapies. Cell Metab. (2014) 20:953–66. 10.1016/j.cmet.2014.09.018 25456737

[50] Zhang CS Li M Ma T Zong Y Cui J Feng JW. Metformin activates AMPK through the lysosomal pathway. Cell Metab. (2016) 24:521–2. 10.1016/j.cmet.2016.09.003 27732831

[51] Skelly PJ Stein LD Shoemaker CB. Expression of *Schistosoma mansoni* genes involved in anaerobic and oxidative glucose metabolism during the cercaria to adult transformation. Mol Biochem Parasitol. (1993) 60:93–104. 10.1016/0166-6851(93)90032-S 8396206

[52] Santos TM Johnston DA Azevedo V Ridgers IL Martinez MF Marotta GB Analysis of the gene expression profile of *S mansoni* cercariae using the expressed sequence tag approach. Mol Biochem Parasitol. (1999) 103:79–97. 10.1016/S0166-6851(99)00100-0 10514083

[53] Roquis D Lepesant JM Picard MA Freitag M Parrinello H Groth M. The epigenome of *Schistosoma mansoni* provides insight about how cercariae poise transcription until infection. PLoS Negl Trop Dis. (2015) 9:e0003853. 10.1371/journal.pntd.0003853 26305466 PMC4549315

[54] Lamb EW Crow ET Lim KC Liang Y Lewis FA Davies SJ. Conservation of CD4+ T cell-dependent developmental mechanisms in the blood fluke pathogens of humans. Int J Parasitol. (2007) 37:405–15. 10.1016/j.ijpara.2006.11.001 17196594 PMC1858658

[55] Mikhail MM Mansour MM. The relationship between serum carnitine levels and the nutritional status of patients with schistosomiasis. Clinica Chimica Acta (1976) 71:207–14. 10.1016/0009-8981(76)90532-5 963890

[56] Coutinho EM. Malnutrition and hepatic fibrosis in murine schistosomiasis. Mem Inst Oswaldo Cruz, Rio de Janeiro (2004) 99:85–92. 10.1590/S0074-02762004000900015 15486641

[57] Mbabazi PS Andan O Fitzgerald DW Chitsulo L Engels D Downs JA. Examining the relationship between urogenital schistosomiasis and HIV infection. PLoS Negl Trop Dis. (2011) 5:e1396. 10.1371/journal.pntd.0001396 22163056 PMC3232194

[58] Sousa-Figueiredo JC Gamboa D Pedro JM Fancony C Langa AJ Magalhaes RJ. Epidemiology of malaria, schistosomiasis, geohelminths, anemia and malnutrition in the context of a demographic surveillance system in northern Angola. PLoS ONE (2012) 7:e33189. 10.1371/journal.pone.0033189 22493664 PMC3320883

[59] Simon GG. Impacts of neglected tropical disease on incidence and progression of HIV/AIDS, tuberculosis, and malaria: scientific links. Int J Infect Dis. (2016) 42:54–7. 10.1016/j.ijid.2015.11.006 26594012

[60] Abbud W Habinowski S Zhang JZ Kendrew J Elkairi FS Kemp BE. Stimulation of AMP-activated protein kinase (AMPK) is associated with enhancement of Glut1-mediated glucose transport. Arch Biochem Biophys. (2000) 380:347–52. 10.1006/abbi.2000.1935 10933890

[61] O'Neill HM. AMPK and exercise: glucose uptake and insulin sensitivity. Diabetes Metab J. (2013) 37:1–21. 10.4093/dmj.2013.37.1.1 23441028 PMC3579147

