# Supplementary material for: Host Adaptive Immune Status Regulates Expression of the Schistosome AMP-Activated Protein Kinase
Source: Front Immunol. 2018 Nov 21;9:2699. doi: 10.3389/fimmu.2018.02699 (PMC6260181; doi:10.3389/fimmu.2018.02699)
Supplement: Supplementary file 1 [file Data_Sheet_1.pdf]

Supplementary Material

Host Adaptive Immune Status Regulates Expression of the Schistosome AMP-Activated Protein Kinase

Running title: Immune Regulation of Schistosome AMPK

Kasandra Hunter and Stephen J. Davies*

Department of Microbiology and Immunology, F. Edward Hébert School of Medicine, Uniformed Services University of the Health Sciences, 4301 Jones Bridge Road, Bethesda, MD 20814, United States of America

* Corresponding author
stephen.davies@usuhs.edu

Supplementary Figure 1. Analysis of the predicted *S. mansoni* AMPK α cDNA

A, The amino acid sequence of a putative *S. mansoni* AMPK α subunit (XP_018653781.1), predicted by the Schistosome Genome Consortium at locus Smp_142990, was aligned with the amino acid sequences of *Homo sapiens* AMPK α-1 (NP_006242) and AMPK α-2 (NP_006243), *Crassostrea gigas* AMPK α-2 (XP_011446066), and *Echinococcus granulosus* AMPK α (AER10553). Sequence identity and similarity are shown, in addition to the approximate locations of the metal-dependent phosphohydrolase (HD_3) domain (CCD accession number pfam13023), AMPK α serine/threonine kinase catalytic domain (STKc, CCD accession number cd14079), AMPK α UBA-like autoinhibitory domain (UBA AID, CCD accession number cd14336), and AMPK α C-terminal regulatory domain (CTRD, CCD accession number cd12122). B, The predicted mRNA (XM_018799915.1) encoded by locus Smp_142990 was aligned with matching ESTs from the NCBI EST database.

A

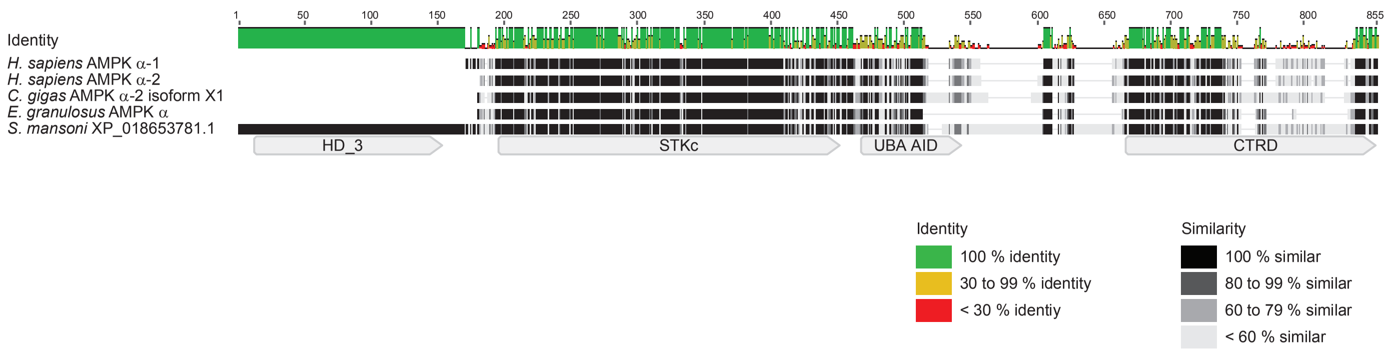
1
50
100
150
200
250
300
350
400
450
500
550
600
650
700
750
800
855
Identity
H. sapiens AMPK α-1
H. sapiens AMPK α-2
C. gigas AMPK α-2 isoform X1
E. granulosus AMPK α
S. mansoni XP_018653781.1
HD_3
STKc
UBA AID
CTRD
Identity
100 % identity
30 to 99 % identity
< 30 % identiy
Similarity
100 % similar
80 to 99 % similar
60 to 79 % similar
< 60 % similar

B

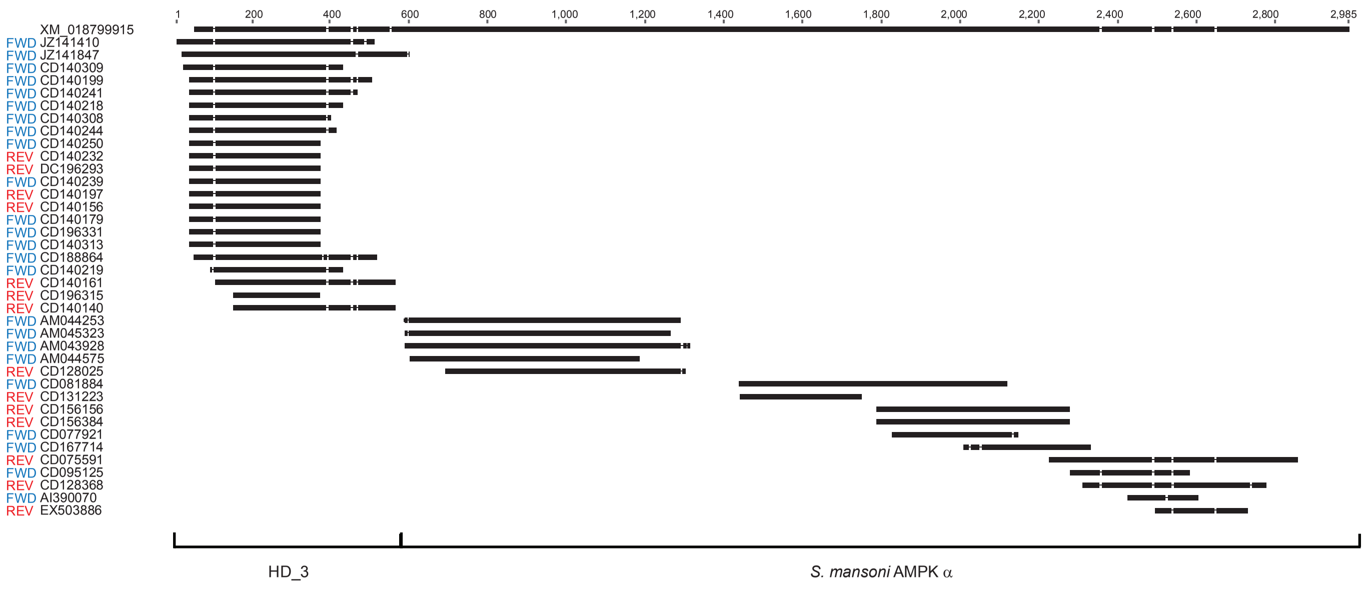
1
200
400
600
800
1,000
1,200
1,400
1,600
1,800
2,000
2,200
2,400
2,600
2,800
2,985
XM_018799915
FWD JZ141410
FWD JZ141847
FWD CD140309
FWD CD140199
FWD CD140241
FWD CD140218
FWD CD140308
FWD CD140244
FWD CD140250
REV CD140232
REV DC196293
FWD CD140239
REV CD140197
REV CD140156
FWD CD140179
FWD CD196331
FWD CD140313
FWD CD188864
FWD CD140219
REV CD140161
REV CD196315
REV CD140140
FWD AM044253
FWD AM045323
FWD AM043928
FWD AM044575
REV CD128025
FWD CD081884
REV CD131223
REV CD156156
REV CD156384
FWD CD077921
FWD CD167714
REV CD075591
FWD CD095125
REV CD128368
FWD AI390070
REV EX503886
HD_3
S. mansoni AMPK α
